# Static and dynamic electro-optical properties of liquid crystals mediated by ferroelectric polymer films

**DOI:** 10.1039/c7ra12443k

**Published:** 2018-01-09

**Authors:** Y. A. Garbovskiy, D. R. Evans, P. P. Banerjee, A. V. Glushchenko

**Affiliations:** UCCS BioFrontiers Center, Department of Physics, University of Colorado Colorado Springs Colorado Springs CO 80918 USA ygarbovs@uccs.edu; Air Force Research Laboratory, Materials and Manufacturing Directorate, Wright Patterson Air Force Base OH 45433 USA; Electro-Optics and Photonics, and Electrical and Computer Engineering, University of Dayton Dayton OH 45469 USA

## Abstract

This paper reports the electro-optical properties of high resistivity nematic liquid crystals sandwiched between ferroelectric polymer films. Interactions between liquid crystals and the film result in a series of interesting optical and electro-optical features. For example, the visualization of ferroelectric domains by means of liquid crystals has been known for decades. However, here we demonstrate that liquid crystals can also reveal the fractal dimension of multi—domain poly(vinylidene fluoride)-based films. Unidirectionally rubbed films made of poly(vinylidene fluoride)-based (PVDF) materials align liquid crystals (LC) homogeneously, with the pretilt angle on the order of 1–2 degrees. This property was implemented in the design of hybrid cells composed of liquid crystals sandwiched between PVDF-based films. The designed PVDF|LC|PVDF cells exhibit tunable electro-optical performance originating from the presence of the PVDF-based films. More specifically, (i) the threshold voltage characterizing the transition of liquid crystals from a planar to a homeotropic state can be tuned by varying the film thickness, and (ii) total fall time (turn-off time) can be controlled by varying the frequency and amplitude of the driving voltage. This frequency dependence of the fall time is strongly pronounced at a relatively high voltage applied across the cell. In the low frequency regime, an increase in the turn-off time can be approximated as a linear function of the applied electric field. An electric-field induced polarization of the PVDF-based films is considered a major reason leading to the afore-mentioned amplitude and frequency dependence of the switching time.

## Abstract 2

Liquid crystals (LC) can reveal the fractal dimension of multi-domain ferroelectric films (FF) while these films can control the switching time of FF–LC hybrids.

## Graphical abstract

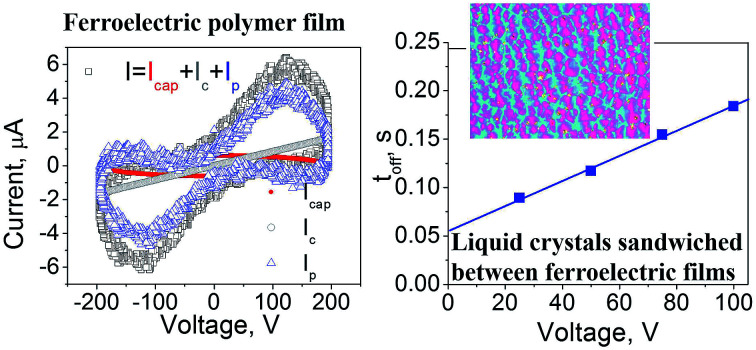

## Introduction

Early studies of the behaviour of liquid crystals on ferroelectric substrates date back to the 1970s.^1–3^ These very first papers reported the visualization of ferroelectric domains of triglycine sulfate (TGS) crystals by observing a thin layer of nematic liquid crystals sandwiched between the cleavage surface of the ferroelectric crystal and a cover glass.^1–3^ The domain visualization is explained considering the dependence of the liquid crystal alignment on the surface tension coefficients characterizing an interface between liquid crystals and substrates.^1–3^ Follow up efforts^4,5^ were directed to study the domain structure of TGS crystals along with anchoring and alignment effects in liquid crystals on the ferroelectric surface.^6,7^ The visualization of ferroelectric domains by means of liquid crystals was also achieved in a variety of inorganic crystals.^8,9,12–14^ In addition to the visualization of the domain structure, the “decoration” of a ferroelectric surface by liquid crystals can also reveal the switching processes (switching dynamics) in ferroelectric crystals.^10–18^ Another interesting application of nematic liquid crystals includes the visualization of stress fields in ferroelectrics.^19^ While in the majority of publications^1–17^ both statics and dynamics of ferroelectric domains are explored using nematic liquid crystals, Ivanov *et al.*^18^ demonstrated a high promise of ferroelectric liquid crystals for this kind of application. More specifically, the use of ferroelectric liquid crystals can overcome shortcomings of nematics such as slow response, high fluidity, and their instability against disclinations.^18^

Since late 1990s focus of research activities on liquid crystals/ferroelectric films gradually shifted towards developing new electro-optical and memory devices utilizing these materials.^20^ A polar asymmetry of the electro-optical response was found in nematic liquid crystals sandwiched between polar (ferroelectric) and non-polar substrates.^20^ This asymmetry was explained considering the permanent electric field originated from the spontaneous polarization of the ferroelectric substrate.^20^ The consideration of the direct influence of the spontaneous polarization of ferroelectric substrate on the electro-optics of liquid crystals is an important aspect of paper.^20^ Interactions of liquid crystals with the spontaneous polarization of the ferroelectric film are further explored in papers^21–25^ reporting local Freedericksz transitions^21,22,24^ and their possible use in electro-optical and memory devices.^23,24^ Local reorientation of liquid crystals governed by the spontaneous polarization of the substrate was observed utilizing inorganic metal oxide ferroelectric crystals such as lead zirconate titanate (PZT),^21–24^ and barium titanate.^25^ Recently, PZT-based ceramic in combination with liquid crystals found their application in the design of tunable micro-lens.^26^

In typical liquid crystal cells with PZT-based substrates, additional alignment layers are needed to align liquid crystalline molecules. Moreover, a PZT-based ceramic is toxic and is not compatible with the concept of flexible electronics relying on polymer materials. Ferroelectric polymers emerged as promising candidates for the design of hybrid cells made of liquid crystals and ferroelectric films.^20,27^ As was reported by several independent groups, ferroelectric polymers such as polyvinylidene fluoride (PVDF) – based materials can be used as an alignment layer for nematic liquid crystals.^27–29^ Alignment and electro-optical properties of twisted nematics sandwiched between PVDF-based films are reported in recent papers.^30,31^ An experimental evidence of the strong impact of the remnant polarization of PVDF-based polymer on the alignment of liquid crystals has been shown for polymer dispersed liquid crystals (PDLC).^32,33^ Results presented in Holländer *et al.*^33^ were obtained using cyanobiphenyl-based liquid crystals characterized by a relatively high concentration of residual ions. As was mentioned in Holländer *et al.*,^33^ these mobile ions dramatically reduced the expected effect due to the screening of the remnant polarization of PVDF-based polymer. Unfortunately, dynamic electro-optical studies (electro-optical time response) of the PDLC system made of the PVDF-based polymer and cyanobiphenyl-based liquid crystals were beyond the scope of these papers.^32,33^ Electro-optical data of twisted nematics sandwiched between PVDF-based films described in afore-mentioned publications^29–31^ were obtained in the low voltage regime (less than 3 V μm^−1^). Thus, intrinsic ferroelectric properties of PVDF-based films and their effects on the electro-optical response of liquid crystals were not demonstrated. To summarize, despite an increasing number of publications,^1–31^ there are practically no data showing how ferroelectric films affect the response time of liquid crystals. Moreover, results presented in the majority of the aforementioned papers were obtained at a single frequency, typically 1 kHz. As shown later in this paper, the choice of driving frequency is very important for revealing the impact of spontaneous polarization of the film on the electro-optics of liquid crystals. Therefore, parameters (frequency, amplitude) used to drive the liquid crystal cell should be selected according to the ferroelectric response of the film.

The major goal of this paper is to provide new insights into the electro-optics of liquid crystals sandwiched between PVDF-based ferroelectric films. More specifically, the work in the paper utilizes high resistivity liquid crystals (to avoid the screening problem), and explores a much broader voltage range (50–100 V μm^−1^) as compared to previous reports. An important aspect of this study is an analysis of both ferroelectric and electro-optical properties over a wide frequency range (0.1 Hz to 100 kHz) to identify the most suitable driving conditions. As shown later in the paper, these conditions can reveal a pronounced effect of the PVDF-based film polarization on the electro-optical response of liquid crystals. In addition, a set of complementary data describing the ferroelectric response of PVDF-based films, alignment of liquid crystals by these films, and electro-optical properties of high resistivity liquid crystals driven by PVDF-based films is presented.

## Materials and methods

### Materials and sample preparation

Poly(vinylidene fluoride-*co*-hexafluoropropylene) (its weight average molecular weight *M*_ω_ = 400 000), abbreviated as PVDF–HFP, and poly(vinylidene fluoride) (*M*_ω_ = 530 000), abbreviated as PVDF, both purchased from Aldrich were dissolved in dimethylformamide (DMF) supplied by Alf a Aesar. To achieve a good dissolution of PVDF and PVDF–HFP in DMF, a hot plate equipped with magnetic stirrer was used. The temperature of the hot plate was set to 50–60 °C, and the weight concentration of ferroelectric polymers was varied from 5 to 20%. The choice of PVDF-based polymers was dictated by their excellent ferroelectric properties^34–36^ and their compatibility with mechanical rubbing technology commonly applied to align liquid crystals.^29–31^

Thin films (0.25–1.0 μm thick) of ferroelectric polymers were obtained by spin-coating them onto ITO glass (2000–4000 rpm; 30 s). A soft bake of spin-coated samples performed at 70 °C for 10 min was followed by a 3 hour long annealing process at 140 °C. A contact profilometer was used to measure the thickness of PVDF-based films. To measure ferroelectric properties of PVDF-based films, a layer of gold (∼50 nm) was deposited on their top surface using DESK V coating system from Denton Vacuum.

To study electro-optical properties of nematic liquid crystals placed between two ferroelectric substrates, a sandwich-like cell was prepared. This cell was made of two pieces of indium tin oxide (ITO) coated glass covered with PVDF-based polymer films which were mechanically rubbed with a velvet cloth. The rubbing direction of top and bottom PVDF-films was anti-parallel. The total thickness of the cell was controlled by spacers (3–6 μm). The prepared empty cell was filled with nematic liquid crystals (TL205); these liquid crystals are known for their high resistivity (on the order of 10^10^ ohm m (ref. 37)) and are practically absent of screening effects caused by mobile ions.^38^

### Experimental methods

Ferroelectric properties of PVDF-based polymer films were measured by means of a conventional experimental technique described in Basun *et al.*^39^ The sample under study (ITO|PVDF|gold or ITO|PVDF–HFP|gold) was connected in a series with a reference resistor. An AC electric field (sinusoidal waveform; 100 mHz to 100 kHz) was applied across the cell, and the current through the reference resistor was measured by means of an oscilloscope. The amplitude of the applied voltage was varied from 0 to 200 V.

The prepared liquid crystal cells (ITO|PVDF|LC|PVDF|ITO and ITO|PVDF–HFP|LC|PVDF–HFP|ITO) were examined using a standard method of polarized light microscopy equipped with CCD camera.^40^ Image processing was performed using ImageJ software. This software is available for free at https://imagej.nih.gov/ij/. The fractal dimension of the image was obtained by applying the box-counting dimension method.^41^

The pretilt angle of liquid crystals sandwiched between PVDF-based films was measured by means of the crystal rotation method.^42,43^

For electro-optical measurements, the liquid crystal cell was placed between two crossed polarizers. The axes of the polarizers were set at 45° with respect to the rubbing direction. AC signals (square wave, 100 mHz to 100 kHz) were applied to the cell. The intensity of the laser beam passing through the cell was measured by a photodiode according to a typical procedure of electro-optical measurements for a uniformly aligned liquid crystal sample.^40,44^

## Results and discussion

### Ferroelectric properties of PVDF-based films

If a voltage *V* is applied across the film, the measured current *I* can be written as a sum of three components, namely, a capacitive current *I*_cap_, a conduction current *I*_c_, and a polarization switching current *I*_p_. This measured current can be expressed as (1):

1 *I* = *I*_cap_ + *I*_c_ + *I*_p_ = *C *d*V*/d*t* + *V*/*R* + *A* d*P*/d*t*

where *C* is the capacitance of the film, *R* is its electrical resistance, *A* is the sample surface area, *P* is the spontaneous polarization of the film, and *t* denotes time.

Using “current–voltage” coordinates and assuming a sinusoidal applied signal, the first term of eqn (1) appears as an ellipse, the second term is typically a straight line, and the third term results in a peak with its maximum positioned around the voltage corresponding to the coercive electric field. Thus, an analysis of the experimental current–voltage dependence can reveal the presence of ferroelectricity in thin ferroelectric films.^45^

**Fig. 1:**
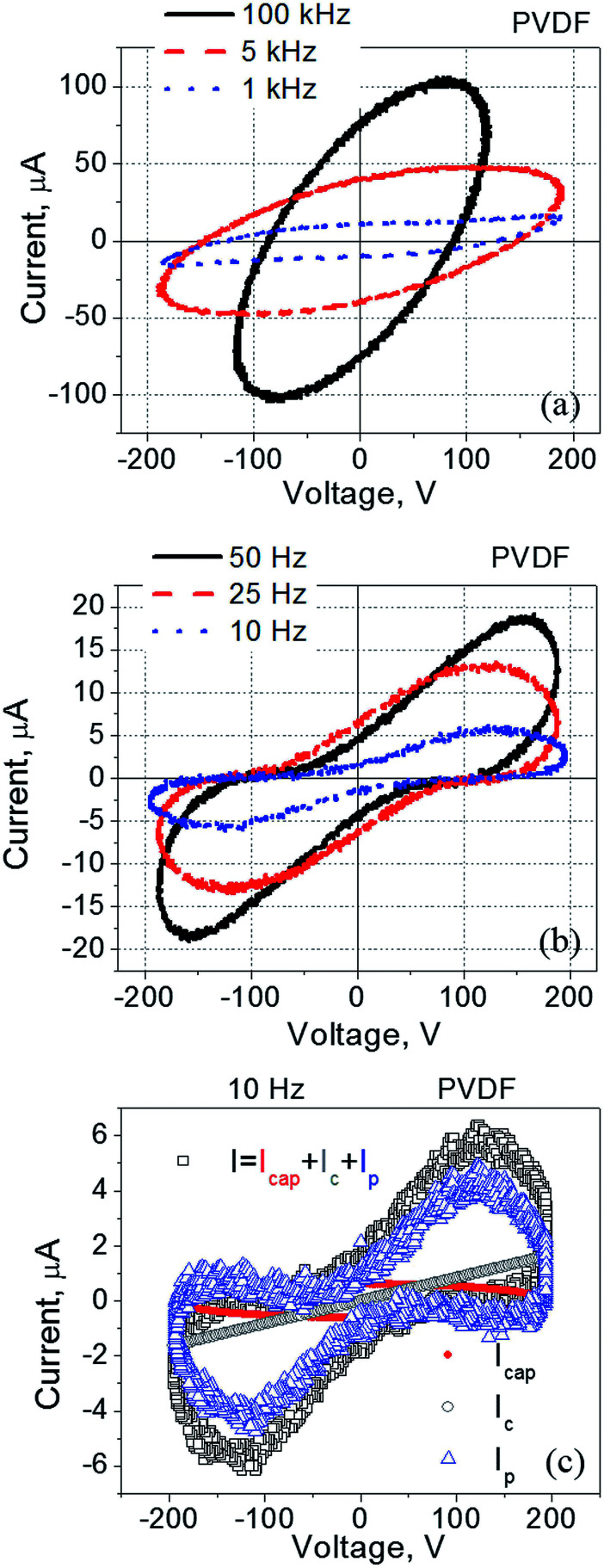
Current–voltage dependence measured for the PVDF-film spin-coated onto ITO coated glass and with a gold electrode deposited on top of the film. The frequency was varied within (a) 1–100 kHz, and (b) 10–50 Hz. (c) The measured current decomposed into three components according to eqn (1).

It was found that current–voltage curves of PVDF-based films exhibit strongly pronounced frequency dependence (Fig. 1). At relatively high frequencies (5–100 kHz) this dependence is represented by a tilted ellipse (Fig. 1a). Thus, the PVDF-based film does not exhibit a measurable ferroelectric response under these conditions (5–100 kHz, with the applied voltage less than 200 V). A decrease in the frequency of the applied electric field dramatically changes the shape of the measured loop (Fig. 1a and b). Upon a frequency reduction, a well pronounced peak appears in the current–voltage dependence indicating the presence of ferroelectric switching at relatively low frequencies (10–25 Hz) (Fig. 1c).

**Fig. 2:**
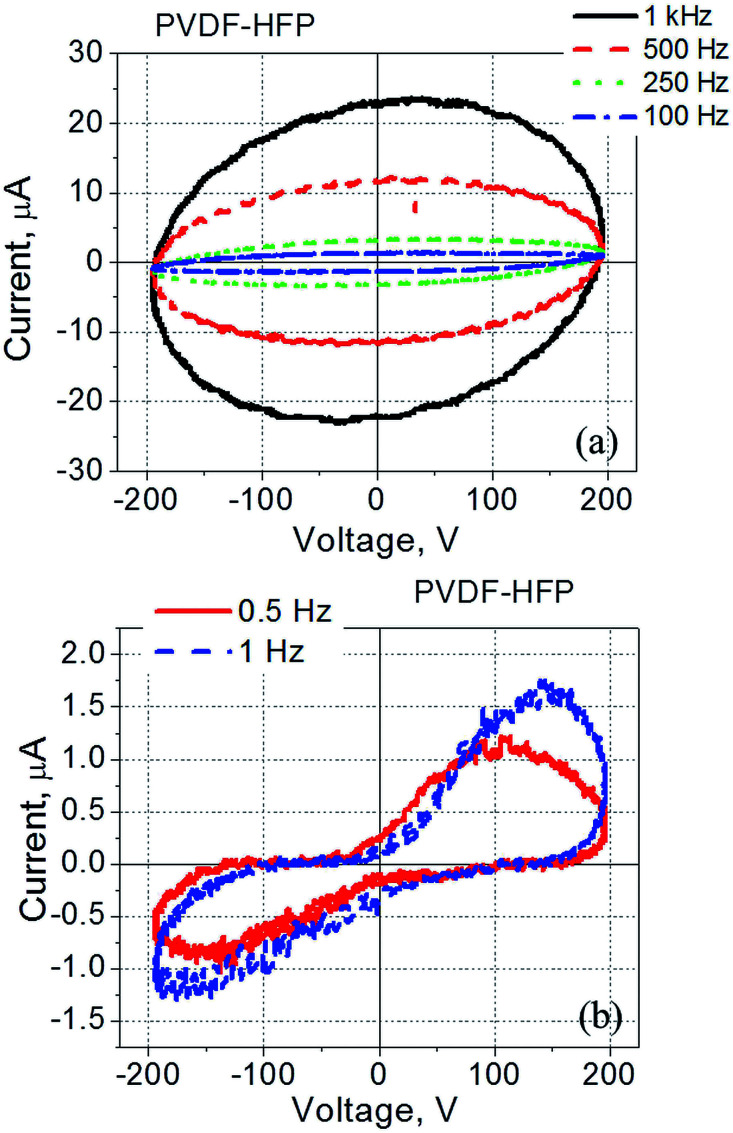
Current–voltage dependence measured for the PVDF–HFP film spin-coated onto ITO coated glass and with a gold electrode deposited on top of the film. The frequency was varied within (a) 100 Hz to 1 kHz, and (b) 0.5–1 Hz.

PVDF–HFP films exhibit similar behaviour (Fig. 2): a response of a typical dielectric in the form of a tilted ellipse at relatively high frequencies (Fig. 2a), and well-pronounced polarization switching current at low frequencies (Fig. 2b).

The observed current–voltage curves shown in Fig. 1 and 2 are in agreement with existing literature reporting the polarization – electric field loops^46,47^ and an increase in the coercive field measured in PVDF-based films at high frequency as compared to the same values at low frequency.^48,49^ For example, by changing frequency from 10 Hz to 100 kHz, the coercive field increased from less than 100 MV m^−1^ to more than 200 MV m^−1^.^48,49^ In regard to the data shown in Fig. 1 and 2, no polarization switching is observed at high frequencies because the applied electric field is below the coercive field, *E* < *E*_c_. At the same time, the polarization switching that takes place at low frequencies resulted from a reduction in the coercive field to a value which is below the applied electric field, *E*_c_ < *E* (Fig. 1b, c and 2b).

Results presented in this section have important practical implications and will be used in the design of the hybrid cell made of liquid crystals and PVDF-based films. More specifically, the use of low frequency electric signals to drive PVDF|LC|PVDF cells allows for the driving voltage reduction. However, in this case liquid crystals should be practically free of ions to avoid ion-related effects such as screening typically observed at low frequency.^38^ As was mentioned above, TL-series nematic liquid crystals are a good choice because of their high resistivity.

### Alignment of liquid crystals by PVDF-based films

As a first step, a visual inspection of the prepared cells (ITO|PVDF|LC|PVDF|ITO and ITO|PVDF–HFP|LC|PVDF–HFP|ITO) under crossed polarizers was performed. Setting the direction of rubbing to coincide with the axis of ether polarizer or analyzer, all samples studied appear dark thus indicating homogeneous planar alignment of TL205 nematic liquid crystals on the PVDF-based films. This finding is in agreement with previous reports.^28–31^

**Fig. 3:**
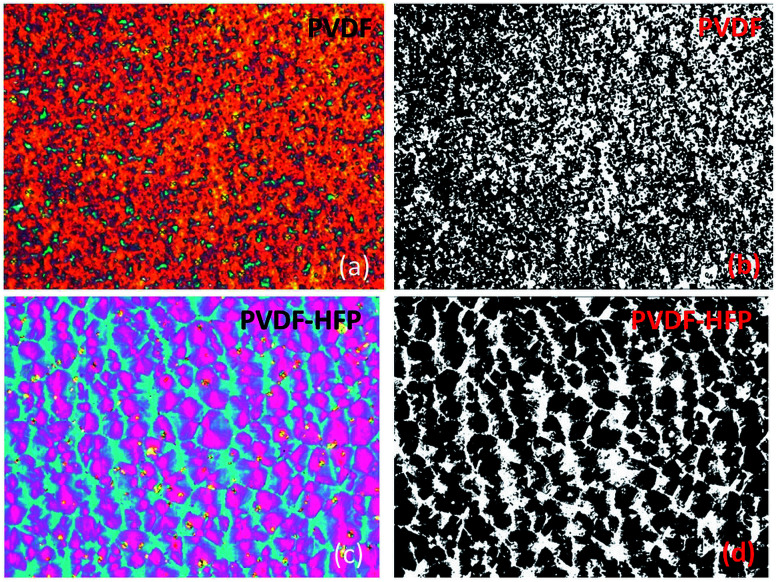
Photomicrographs of the TL205 nematic liquid crystals layer placed between PVDF-based films. The field of view is ∼360 μm× 270 μm. Polarizers are crossed. (a and c) Color images, the rubbing direction is along the diagonal. (b and d) Binary images obtained by processing images (a) and (c), respectively. The thickness of the liquid crystal layer is ∼4.75 μm, and the thickness of the PVDF-based film is ∼1 μm per substrate. The obtained fractal dimension: *D* = 1.8271 (PVDF), and *D* = 1.8718 (PVDF–HFP).

However, further analysis of the prepared samples by means of polarized light microscopy revealed their rather interesting microstructure (Fig. 3). Liquid crystals placed between PVDF-based films appear as two-colored plates with a distinct surface distribution of colors (Fig. 3a and c). This surface distribution of colors can be associated with the multidomain structure of PVDF-based films. An interesting feature of the colored domains shown in Fig. 3a and c is their fractal-like structure. Additional analysis using the box-counting dimension method (Fig. 3b and d) revealed a fractal dimension *D* of the studied samples calculated using the ImageJ software according to eqn (2):2
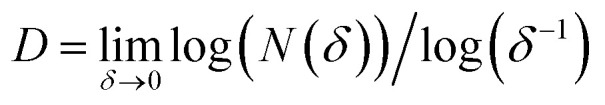
where *N*(*δ*) is the number of boxes characterized by its length *δ* needed to cover a fractal object under study.^41^

The obtained values of the fractal dimension (*D* = 1.8271 (PVDF|TL205|PVDF) and *D* = 1.8718 (PVDF–HFP|TL205|PVDF–HFP)) indicate a high degree of roughness of the boundary between neighboring domains in PVDF-based films. It should be noted that a fractal dimension, 1 ≤ *D* ≤ 2 is typical for thin ferroelectric films.^50–52^ To date, the piezoresponse force microscopy is a tool of choice to measure the fractal dimension of ferroelectric domains.^50–52^ To the best of our knowledge, this paper is a first demonstration of how to measure the fractal dimension of ferroelectric films by means of liquid crystals.

The presence of two dominant colors in the micrographs shown in Fig. 3 requires an additional discussion. Since liquid crystals are uniformly aligned along the rubbing direction of PVDF-based films, the change in color corresponds to the change in the birefringence. If the thickness of the liquid crystal cell is known, the birefringence of the colored section can be determined using the Michel-Levy and Raith-Sorensen interference color charts.^53,54^ By comparing the observed colors to those of the Michel-Levy and Raith-Sorensen charts, the birefringence of pink and blue regions (PVDF–HFP|TL205|PVDF–HFP, Fig. 3) was estimated as 0.199 and 0.232, respectively. Determined values of the effective birefringence are close to the magnitude of the optical birefringence of TL205 liquid crystals, Δ*n* = *n*_e_ − *n*_o_ (*n*_o_ is the ordinary refractive index, and *n*_e_ is the extraordinary refractive index) in the red Δ*n* (*λ* = 630 nm) = 0.200 and in the blue Δ*n* (*λ* = 440 nm) = 0.233, respectively. Using an approximation of uniaxial crystals, the relationship between the optical birefringence, Δ*n*, and the effective birefringence, Δ*n*(*φ*), is expressed by eqn (3) and (4):

3 Δ*n*(*φ*) = *n*(*φ*) − *n*_o_

**Fig. 4:**
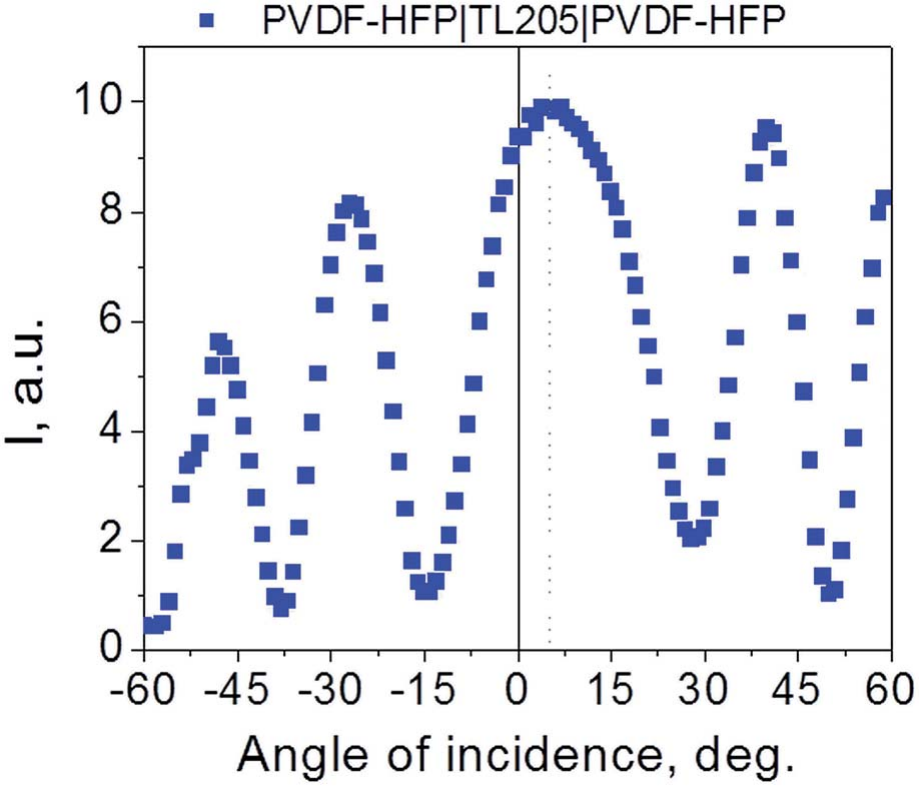
The crystal rotation method: the transmittance of the PVDF–HFP|TL205|PVDF–HFP sample as a function of incident angle. Parameters used to deduce the pretilt angle: the cell thickness is 30 μm; *n*_o_ = 1.519, *n*_e_ = 1.724.

4
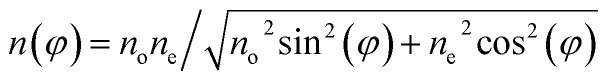
where *φ* is an angle between the wave vector and an optical axis of the uniaxial crystal (in the case of homogeneously aligned nematic liquid crystals it generally coincides with the rubbing direction).^55^ Since the effective optical birefringence (0.199 and 0.232) is very close to the value of the optical birefringence at red and blue wavelength (0.200 and 0.233), the pretilt angle, Θ = 90° − *φ*, is small, on the order of 1–2° (a smaller value of the pretilt corresponds to a higher value of the effective birefringence). Thus, the observed difference in colors shown in Fig. 3 is caused by small variations of the pretilt angle and by the dispersion of the refractive index. This conclusion is supported by direct measurements of the pretilt angle by means of the crystal rotation method. The size of the laser beam (∼1 mm) is more than 100-fold greater than the domain size (Fig. 3). As a result, the measured value of the pretilt angle should be treated as an average quantity. The obtained value of the pretilt angle (1.6° (sample – PVDF–HPF|TL205|PVDF–HPF), Fig. 4) is in agreement with the aforementioned conclusion regarding the colors of the studied samples (Fig. 3). Similar value (1.5°) of the pretilt angle was also measured for the PVDF|TL205|PVDF sample.

### Electro-optical response of liquid crystals placed between PVDF-based films

If a thickness of the PVDF-based film (∼1 μm) is comparable to that of the liquid crystal layer (∼4 μm), the applied electric voltage, *V* can be written as a sum of the voltage across the film, *V*_F_, and the voltage across the liquid crystal, *V*_LC_, according to eqn (5)–(7):^21^

5 *V* = *V*_F_ + *V*_LC_

6 *V*_F_ = *V*/(1 + *d*_LC_*ε*_F_/*d*_F_*ε*_LC_)

7 *V*_LC_ = *V*/(1 + *d*_F_*ε*_LC_/*d*_LC_*ε*_F_)

where *ε*_LC_, *d*_LC_ and *ε*_F_, *d*_F_ are the dielectric permittivity and thickness of liquid crystals and PVDF-based film, respectively.

**Fig. 5:**
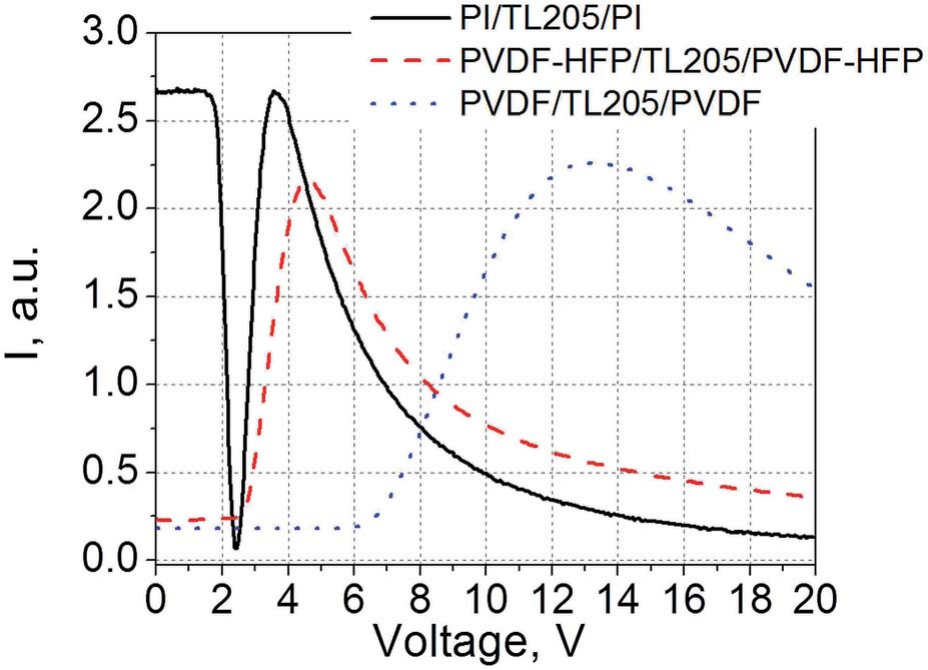
Static electro-optical response of TL205 liquid crystals sandwiched between three different films: PVDF–HFP films (dashed curve); PVDF-films (dotted curve); and polyimide films (PI, solid curve). Measurements are taken at 1 kHz.

An example of static electro-optical response of liquid crystals sandwiched between PVDF-based films is shown in Fig. 5. For comparison, a standard liquid crystal cell with glass substrates coated with polyimide alignment layers was used.

As a result of the voltage redistribution (eqn (5)–(7)), only the voltage *V*_LC_ drives liquid crystals. A voltage applied across the PVDF-based film results in the apparent increase in the threshold voltage (2.5 V for the PVDF–HFP|TL205|PVDF–HFP cell (Fig. 5, dashed curve), and 6 V in the case of PVDF|TL205|PVDF cell (Fig. 5, dotted curve)). A substantial voltage drop across the PVDF-based films is caused by their relatively large thickness (on the order of 1 μm). At the same time, standard cells with nearly 100 nm thick polyimide alignment layers exhibit static electro-optical response typical for TL205 liquid crystals with a threshold voltage around 1.8 V (Fig. 5, solid curve). In this case, *d*_F_ ≪ *d*_LC_ thus *V*_LC_ → *V*.

It should be noted that we use thicker PVDF-based films intentionally in order to see whether the polarization of these films can affect the electro-optical response of liquid crystals. According to eqn (5)–(7), the thicker the thickness of the film, the higher is the voltage drop across it. The electric field needed to observe the polarization switching in PVDF-based films is relatively high (on the order of 50–100 V μm^−1^) and depends on the frequency (Fig. 1 and 2 and ref. 34–36, 48 and 49). A relatively low electric field (∼1–2 V μm^−1^) is required to achieve full reorientation (from a planar to homeotropic state) of nematic liquid crystals with positive dielectric anisotropy (Fig. 5 and ref. 56).

**Fig. 6:**
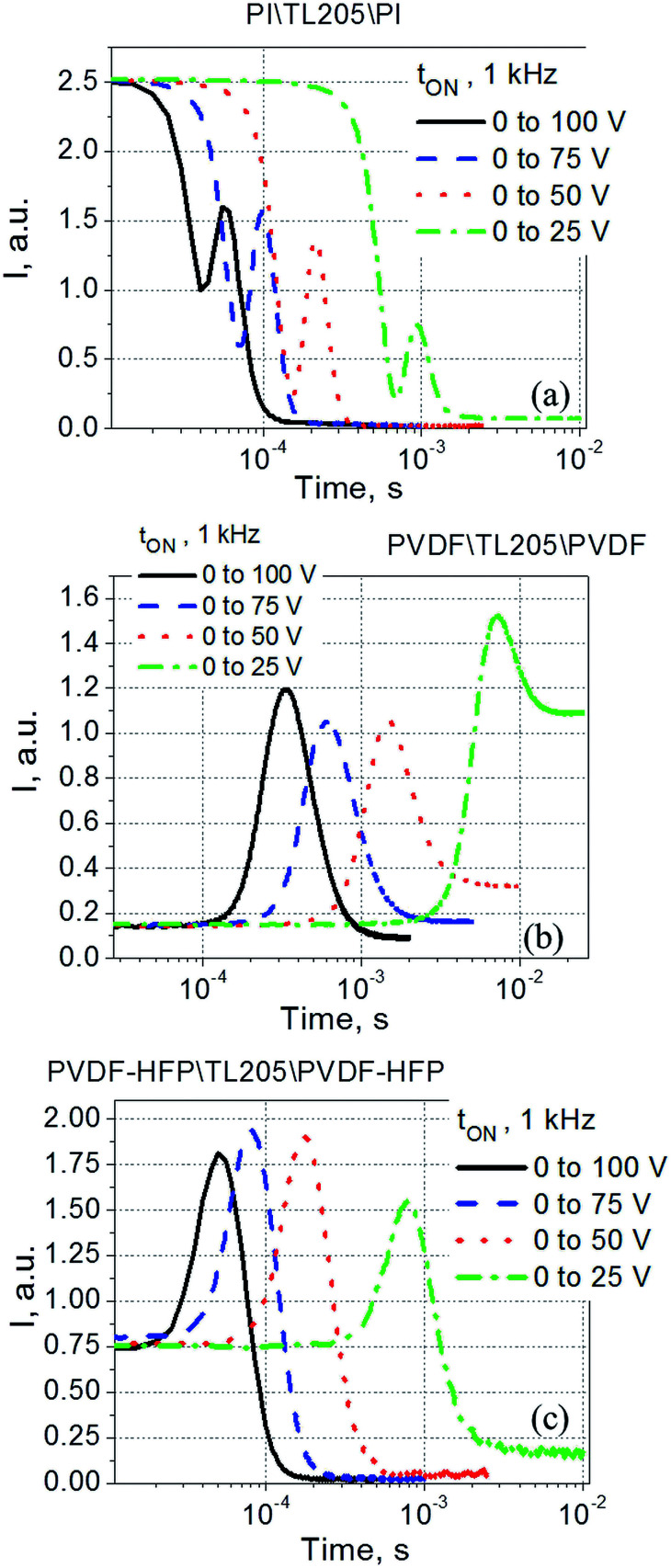
Time response (turn-on time) of TL205 liquid crystals placed between three different films ((a) polyimide films; (b) PVDF-films; and (c) PVDF–HFP films) measured at various values of the applied voltage *V*.

To study the switching time of liquid crystals placed between PVDF-based films, both total rise time (turn-on time, abbreviated as *t*_ON_) and fall time (turn-off time, abbreviated as *t*_OFF_) were measured. The measured data were compared against similar data obtained for the same liquid crystals placed between standard polyimide alignment layers. It was found that the turn-on time of the samples studied did not depend on the frequency of the driving voltage (within the 1–1000 Hz range). An example of typical electro-optical data measured at 1 kHz is shown in Fig. 6.

Experimental data shown in Fig. 6, plotted as *t*_ON_^−1^*versus V*^2^ follow typical for nematic liquid crystals dependence expressed by eqn (8):^56^

**Fig. 7:**
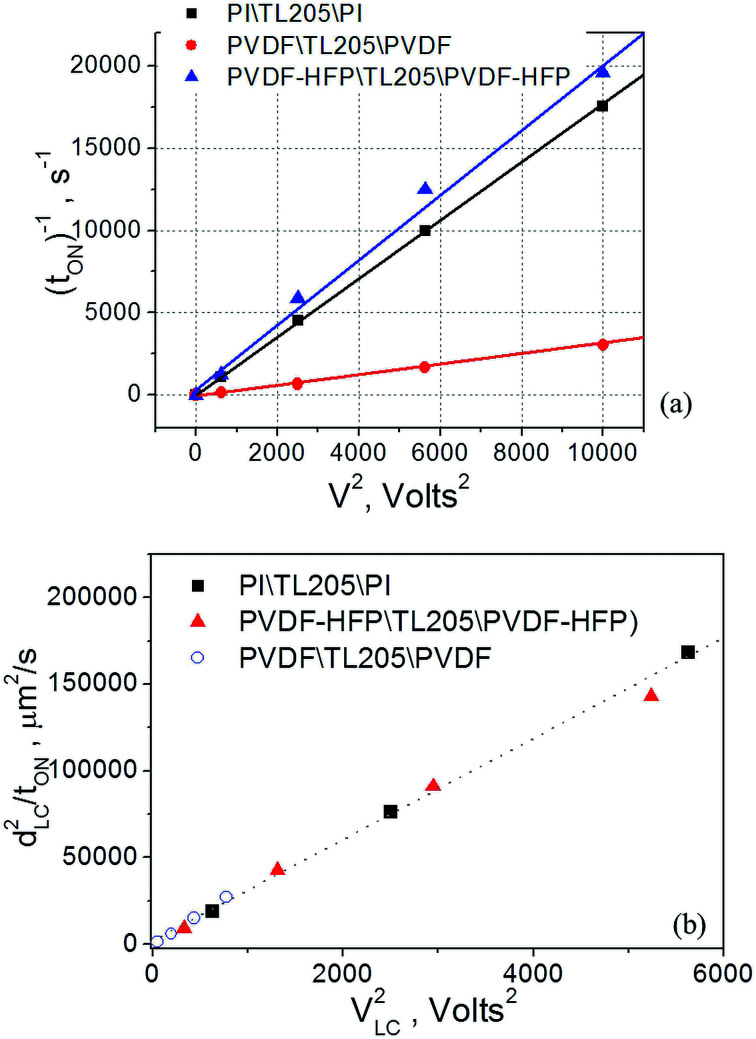
(a) The dependence “*t*_ON_^−1^*versus V*^2^”. (b) The dependence “*d*_LC_^2^*t*_ON_^−1^*versus V*_LC_^2^”. TL205 liquid crystals placed between three different films: polyimide films (square), PVDF-films (circle), and PVDF–HFP films (triangles).

8 *t*_ON_ = *γd*_LC_^2^/(*ε*_o_|Δ*ε*_LC_|*V*_LC_^2^ − π^2^*K*_*ii*_)

where *γ* is the rotational viscosity; *ε*_0_ is an electric constant; Δ*ε*_LC_ is the dielectric anisotropy; and *K*_*ii*_ is an elastic constant (Fig. 7a).

**Fig. 8:**
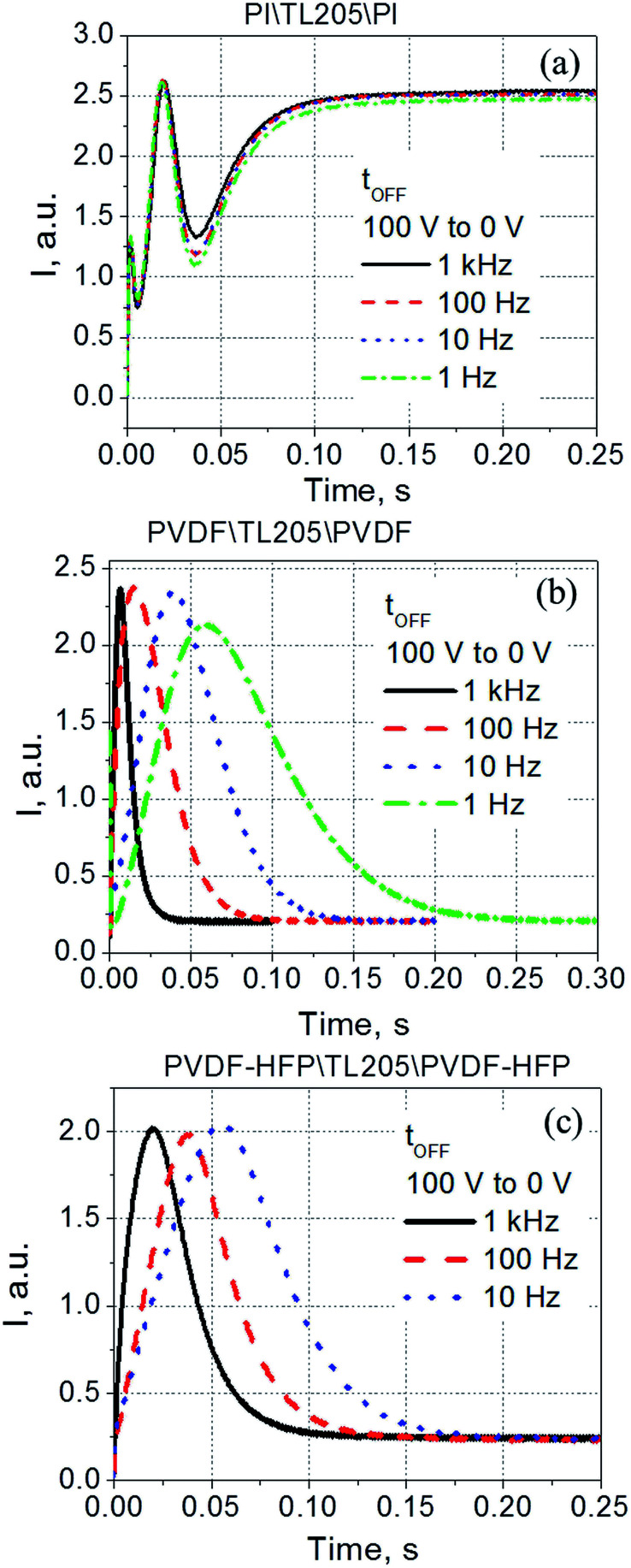
Time response (turn-off time) of TL205 liquid crystals sandwiched between three different films ((a) polyimide films; (b) PVDF-films; and (c) PVDF–HFP films) measured at various values of the frequency of the applied voltage *V*. The amplitude of the applied voltage is fixed (100 V).

According to eqn (5)–(7), the voltage drop across the liquid crystal layer is proportional to the total voltage applied across the sample, *V*_LC_ ∝ *V*. Because *V*_LC_ and *d*_LC_ of the studied samples are not the same, there are three straight lines governed by the same dependence (8) (Fig. 7a). To show more clearly that PVDF-based films practically do not affect turn-on time of liquid crystals, experimental data points were also plotted in “*d*_LC_^2^*t*_ON_^−1^*versus V*_LC_^2^” coordinates (Fig. 7b). In this case, experimental data points of all measured sample are well represented by the same straight line (Fig. 7b). In contrast to the turn-on time, turn-off time of the same samples exhibit a strong frequency dependence. This dependence is illustrated by Fig. 8.

**Fig. 9:**
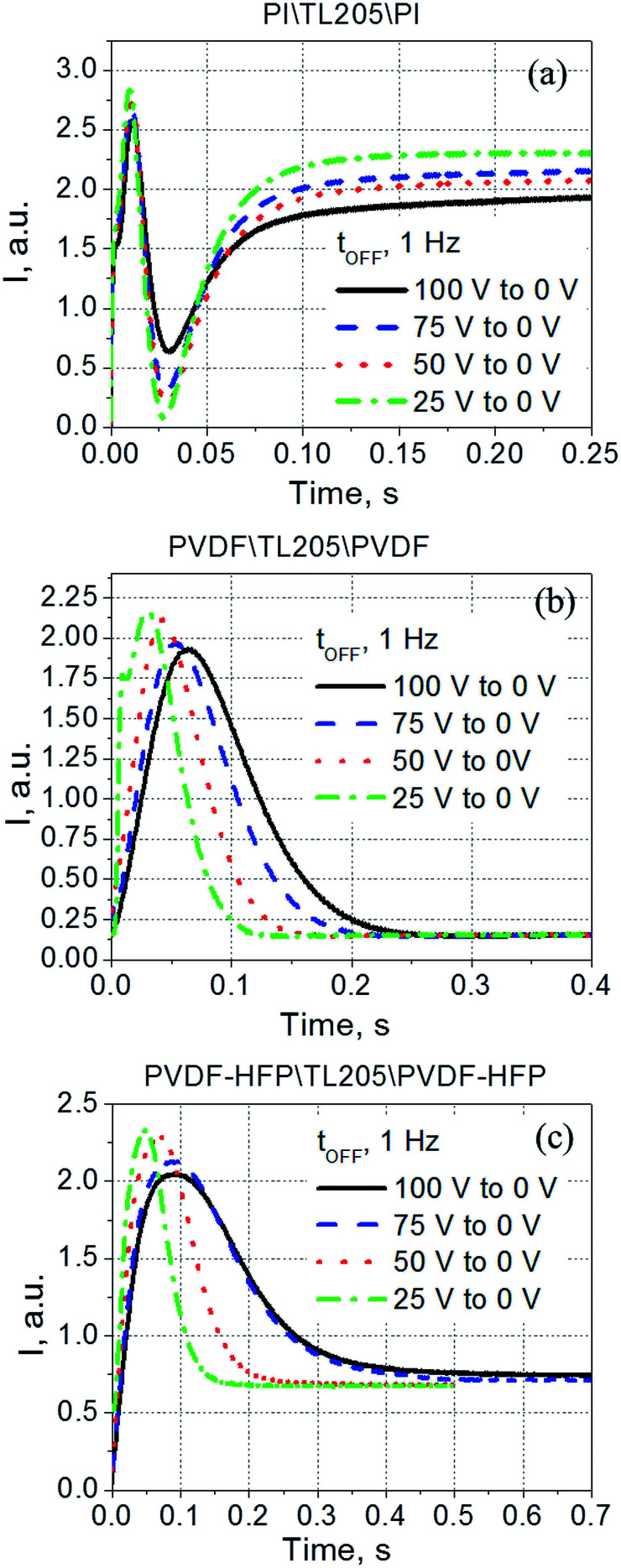
Time response (turn-off time) of TL205 liquid crystals placed between three different films ((a) polyimide films; (b) PVDF-films; and (c) PVDF–HFP films) measured at various values of the applied voltage *V*. The frequency of the applied voltage is fixed (1 Hz).

Contrary to the case of the standard liquid crystal cell with polyimide alignment layer exhibiting frequency independent time response (Fig. 8a), the turn-off time of liquid crystals sandwiched between PVDF-based films increases with decreasing frequency (Fig. 8b and c). This increase in the turn-off time can be very substantial, for example, by changing the frequency from 1 kHz to 1 Hz, the turn-off time increases more than 7 fold, from less than 30 ms to nearly 220 ms (Fig. 8b). Further studies of this interesting behaviour revealed its dependence on the amplitude of the applied voltage (Fig. 9). It should be noted that turn-off time of standard liquid crystal cell with polyimide alignment layers does not depend on the amplitude of the applied voltage (assuming this voltage is high enough to completely reorient the molecules from a planar to homeotropic state) as is shown in Fig. 9a. The turn-off time of liquid crystals sandwiched between PVDF-based films increases with increasing applied voltage (Fig. 9b and c). For example, by increasing the amplitude of the applied low-frequency voltage from 25 V to 100 V, the turn-off time of TL205 liquid crystals sandwiched between PVDF-based films increases more than 2-fold: from ∼0.1 s to ∼0.23 s (PVDF|TL205|PVFD, Fig. 9b), and from ∼0.15 s to ∼0.4 s (PVDF–HFP|TL205|PVDF–HFP, Fig. 9c). A reasonable qualitative explanation of the observed electro-optical behaviour (Fig. 8 and 9) can be proposed by considering a strong dependence of the ferroelectric properties of PVDF-based films on both amplitude and frequency of the applied voltage (Fig. 1 and 2). The applied low-frequency voltage of increasing amplitude can polarize the PVDF-based film. The polarization of the film increases with increasing voltage and this effect becomes much more pronounced at low frequencies. If the applied voltage is turned OFF, the electric field of the polarized PVDF-based film has a non-zero value since it does not vanish instantaneously. This electric field tends to keep liquid crystal molecules in a homeotropic state. However, because of the natural relaxation of the induced polarization of the PVDF-based film, liquid crystal molecules gradually reorient from homeotropic to their initial planar state. As a result, the polarized PVDF-based film effectively increases the turn-off time of liquid crystals making it dependent on both the amplitude and the frequency of the applied voltage (Fig. 8 and 9). An analysis of experimental data shown in Fig. 9a–c indicates that the measured turn-off time can be approximated by the following empirical eqn (9):

9 *t*_OFF_ ≈ *t*^0^_OFF_ + Δ*t*_OFF_

where the first term of the equation, *t*^0^_OFF_, is an intrinsic turn-off time (measured for liquid crystals placed between standard “non-polarized” polyimide alignment layers on glass substrates); and the second term, Δ*t*_OFF_, is an increase in the turn-off time caused by the polarization of the PVDF-based film. An intrinsic turn-off time is typically expressed by eqn (10):^56^

10 *t*^0^_OFF_ = *γd*^2^_LC_/*K*_*ii*_π^2^

**Fig. 10:**
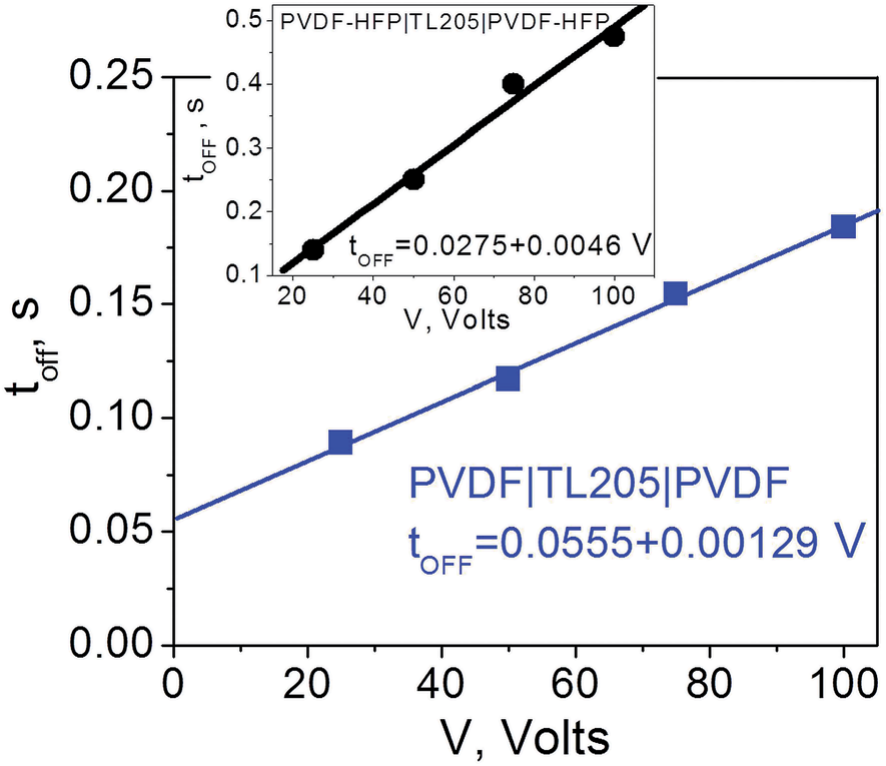
Time response (turn-off time) of TL205 liquid crystals placed between PVDF-films (filled squares) and PVDF–HFP films (filled circles) as a function of the applied voltage. The frequency of the applied voltage is fixed (1 Hz). Straight lines show theoretical fit according to eqn (9) where Δ*t*_OFF_ ∝ *V*.

As can be seen from Fig. 10, an increase in the turn-off time, Δ*t*_OFF_, can be approximated as a linear function of the applied voltage, Δ*t*_OFF_ ∝ *V*.

The observed linear dependence of the turn-off time for the PVDF|TL205|PVDF cells on the applied voltage (Fig. 10) suggests an asymptotic power law to describe the relaxation of the polarization *P*(*t*) induced in PVDF-based films by the applied voltage:^57^

11 *P*(*t*) = *P*_0_(*t*/*τ*)^−1^

where *P*_0_ is the polarization of the PVDF-based film induced by the applied electric field prior to turning the field off; *τ* is the characteristic time corresponding to the relaxation of the microscopic structural unit of the PVDF-based films; *t* is time. Since the measured turn-off time is much greater than the intrinsic time, *t*_OFF_ ≫ *t*^0^_OFF_, it can be estimated using eqn (11) and assuming a quasi-linear dielectric response of the PVDF-based films (the applied electric field is below the coercive field). In this case *P*_0_ ∝ *V*, *t* ≈ Δ*t*_OFF_, and *P*(Δ*t*_OFF_) ≈ *P*_th_. The polarization *P*_th_ corresponds to the electric field associated with the Freedericksz transitions in TL205 nematic liquid crystals. By plugging these quantities into eqn (11), we can obtain the linear dependence, Δ*t*_OFF_ ∝ *Vτ*/*P*_th_, experimentally observed in this paper (Fig. 10).

## Conclusions

The combination of nematic liquid crystals and ferroelectric polymer films results in new features of optical and electro-optical behaviour observed in such materials. The visualization of domains in ferroelectric crystals by decorating them with liquid crystals has been known since the early 1970s. In this paper, we have shown that the domain visualization originates from small variations of the pretilt angle (on the order of 1–2°) of liquid crystals. In addition, it has also been demonstrated that liquid crystals can reveal the fractal dimension of ferroelectric polymer crystals (Fig. 3). To the best of our knowledge, this is a first demonstration of how to measure the fractal dimension of ferroelectric films by means of liquid crystals. This finding has an important practical application since it can complement existing methods such as piezoresponse force microscopy to study multi-domain structure of ferroelectric crystals.

To design a hybrid electro-optical cell composed of nematic liquid crystals sandwiched between mechanically rubbed ferroelectric polymer films, a special consideration should be given to the choice of driving voltage (waveform, amplitude and frequency). We have demonstrated that low frequency signals (1–10 Hz) of high amplitude (100–200 V) applied across ∼1 μm thick PVDF-based film resulted in the polarization switching current, whereas voltage of the same amplitude and higher frequency (100–10 000 Hz) led to a response of a typical lossy capacitor (Fig. 1 and 2). That is why thicker PVDF-based films and thinner liquid crystal layers (the film thickness should be comparable to the thickness of the liquid crystals layer) in combination with low frequency voltage of relatively high amplitude are very advantageous for the design of the afore-mentioned hybrid cells.

The voltage redistribution between the PVDF-based films and liquid crystals resulted in the apparent increase in the threshold voltage of the Freedericksz transitions characterizing the static electro-optical response of these materials. This threshold voltage increases from 1.8 V (TL205 liquid crystals placed between polyimide alignment layers) to 2.5 V (the same liquid crystals sandwiched between rubbed PVDF–HFP films) and 6 V (TL205 liquid crystals sandwiched between rubbed PVDF-films) (Fig. 5).

The voltage applied across the PVDF|LC|PVDF and PVDF–HFP|LC|PVDF–HFP cells polarized the PVDF-based film. At the same time, it also reoriented the liquid crystal molecules from a planar to homeotropic state. We found that time needed to complete this process (turn-on time) was not altered by the presence of the PVDF-based films (Fig. 6 and 7) and followed a standard dependence expressed by eqn (8). In contrary, the relaxation time (turn-off time) describing the reorientation of liquid crystal molecules from a homeotropic to planar state upon turning off the applied voltage was strongly affected by the PVDF-based films (Fig. 8 and 9). This turn-off time depended on the amplitude of the applied voltage and became much more pronounced at lowering the frequency of the voltage. In the regime of low frequency, the measured turn-off time of liquid crystals sandwiched between PVDF-based films can be expressed as a sum of two components, *t*_OFF_ = *t*^0^_OFF_ + Δ*t*_OFF_ (eqn (9)). The first term of this equation accounts for the intrinsic turn-off time of liquid crystals expressed by eqn (10), and the second term describes an increase in the turn-off time of liquid crystals caused by their interactions with PVDF-based films. We found that this increase was a linear function of the applied voltage, Δ*t*_OFF_ ∝ *V* (Fig. 10). An electric field of the polarized PVDF-based film with the polarization relaxation following an asymptotic power law (eqn (11)) was proposed as a major reason leading to the observed increase in the turn-off time, Δ*t*_OFF_ ∝ *V*.

## Conflicts of interest

There are no conflicts to declare.

## Supplementary Material
